# Labor in pregnant women with heart disease

**DOI:** 10.1590/1806-9282.20251060

**Published:** 2025-12-05

**Authors:** Felipe Favorette Campanharo, Edward Araujo, Rosiane Mattar

**Affiliations:** 1 Universidade Federal de São Paulo, Escola Paulista de Medicina, Department of Obstetrics – São Paulo (SP), Brazil.

A cardiological approach to labor for pregnant women with heart disease should optimize the patient's functional status before conception. Once pregnant, it is essential to receive prenatal follow-up care from specialized services with multidisciplinary and interprofessional teams. Counseling patients and monitoring them for signs of clinical decompensation is also important^1^.

From an obstetric point-of-view, the best time and route of delivery must be discussed for each patient. Adequate maternal–fetal monitoring is necessary during labor, as well as attention to water balance. In the puerperium, medications should be reevaluated and adjusted^2^.

Clinical guidelines for the route of delivery in pregnant women with heart disease are largely based on expert opinion. These guidelines reaffirm that, in women with adequate cardiac output, vaginal delivery is preferred because it is associated with less blood loss, faster postpartum recovery, and lower thrombotic and infectious risks. However, a cesarean section could be a better option for selected patients. Some pregnant women with heart disease have indications for a cesarean section, as the risks to the mother and fetus would be greater during labor (Table 1)^3^.

**Table 1:** Indications for cesarean section in pregnant women with heart disease.

Severe heart failure
Aortic root ≥45 mm—when the aorta 40–45 mm, individualize cases of aortic dissection
Severe forms of pulmonary hypertension—including Eisenmenger's syndrome
Labor while on oral anticoagulants

The incidence of cesarean section among pregnant women with heart disease ranges from 21 to 55% worldwide^3^. In Brazil, the cesarean section rate among pregnant women with heart disease has reached an alarming 76%^4^. However, it seems that the occurrence of adverse outcomes after vaginal delivery and cesarean section is similar^3^. It has also been found that maternal outcomes are significantly related to the nature of the cardiac lesion rather than the route of delivery. Cardiac complications can occur during pregnancy, delivery, and the postpartum period (Table 2)^5^.

**Table 2:** "Feared" intrapartum events in pregnant women with heart disease.

Cardiac events in the intrapartum
Heart failure—arrhythmias—aortic dissection—acute pulmonary edema
Obstetric events in pregnant women with heart disease
Need for induction/inhibition of labor Pre-eclampsia—postpartum hemorrhage—thromboembolic phenomena

For pregnant women with heart disease, induction of labor at 40 weeks should be considered, as the benefits outweigh the risks. Misoprostol (PGE1) and dinoprostone (PGE2) are both effective in preparing the cervix and inducing labor^6^. Methods such as the Krause balloon, amniotomy, and oxytocin infusion are also considered safe^7^. However, the inhibition of preterm labor should be approached with caution since most drugs used in tocolysis have cardiovascular effects. Consider the risk of decompensation versus the risk of prematurity, and when indicated, combine with corticosteroids to promote fetal lung maturation^8^.

Regarding tocolytic agents, nifedipine can induce hypotension and shows synergism when used with magnesium sulfate. Terbutaline has intense β-mimetic effects, which can lead to cardiac decompensation^9^. Atosiban is a competitive antagonist of the human oxytocin receptor. In animals, atosiban showed no cardiovascular effects, making it the preferred medication in these situations. It is administered via intravenous infusion of approximately 400 mL of solution (0.9% saline, Ringer's, or 5% dextrose) over 48 h (approximately 200 mL/24 h)^10^.

Special attention should be given to delivery in anticoagulated pregnant women. Ideally, their delivery should be "planned" at around 39 weeks of gestation to avoid hemorrhagic risks. In cases of high thrombotic risk when the pregnant woman is using low-molecular-weight heparin (LMWH), switch to unfractionated heparin around 36 h before delivery. Stop the infusion 4–6 h before birth. Activated partial thromboplastin time levels should guide therapy. Low-risk women using therapeutic or prophylactic LMWH—the usual twice-daily regimen—should omit the nighttime dose and undergo labor induction or a cesarean section the following morning. Loco-regional blockades are possible if more than 24 h have elapsed since the last dose. Remember that this population is at risk of postpartum hemorrhage, so active management is recommended in the third stage of labor^11^.

In conclusion, labor and delivery in pregnant women with heart disease require individualized planning, multidisciplinary care, and careful evaluation of maternal and fetal risks. While vaginal delivery is often preferred, cesarean section may be indicated in specific cardiac conditions. Understanding the unique challenges and applying evidence-informed protocols can reduce maternal morbidity and improve perinatal outcomes in this high-risk population.

## Data Availability

The datasets generated and/or analyzed during the current study are available from the corresponding author upon reasonable request.
